# Constitutively altered frequencies of circulating follicullar helper T cell counterparts and their subsets in rheumatoid arthritis

**DOI:** 10.1186/s13075-014-0500-6

**Published:** 2014-12-05

**Authors:** Irene Arroyo-Villa, María-Belén Bautista-Caro, Alejandro Balsa, Pilar Aguado-Acín, María-Gema Bonilla-Hernán, Chamaida Plasencia, Alejandro Villalba, Laura Nuño, Amaya Puig-Kröger, Emilio Martín-Mola, María-Eugenia Miranda-Carús

**Affiliations:** Department of Rheumatology, Hospital Universitario La Paz-IdiPaz, Paseo de La Castellana, 261, 28046 Madrid, Spain; Laboratorio de Inmuno-Oncología, Hospital General Universitario Gregorio Marañón, Calle Doctor Esquerdo, 46, 28007 Madrid, Spain

## Abstract

**Introduction:**

Circulating CD4 T cells expressing CXCR5, ICOS and/or PD-1 are counterparts of follicular helper T cells (Tfh). There are three subpopulations of circulating Tfh (cTfh): CXCR5 + CXCR3 + CCR6- (Tfh-Th1), CXCR5 + CXCR3-CCR6- (Tfh-Th2) and CXCR5 + CXCR3-CCR6+ (Tfh-Th17). Our objective was to study the B cell helping capacity of cTfh subsets, and examine their frequency in Rheumatoid Arthritis (RA) patients, together with the frequency of circulating plasmablasts (CD19 + CD20-CD38^high^).

**Methods:**

Peripheral blood was drawn from RA patients with active disease (RA-a, DAS28 >2.6) (n = 17), RA in remission (RA-r, DAS28 <2.6) (n = 17) and healthy controls (HC) (n = 34). cTfh and plasmablast frequencies were determined by flow cytometry. Cocultures of sorted CD4 + CXCR5+ T cell subpopulations were established with autologous CD19 + CD27- naïve B cells of HC, and concentrations of IgG, A and M were measured in supernatants.

**Results:**

Isolated Tfh-Th2 and Tfh-Th17 but not Tfh-Th1 cells, induced naïve B cells to secrete IgG and IgA. The frequency of CXCR5+ cells gated for CD4+ T cells was not different among HC, RA-a and RA-r. In contrast, both RA-a and RA-r patients demonstrated an increased frequency of CD4 + CXCR5 + ICOS+ T cells and augmented (%Tfh-Th2 + %Tfh-Th17)/%Tfh-Th1 ratio as compared with HC. In addition, RA-a but not RA-r patients, showed an increased frequency of circulating plasmablasts.

**Conclusion:**

Both RA-a and RA-r patients demonstrate an increased frequency of cTfh and overrepresentation of cTfh subsets bearing a B cell helper phenotype, suggesting that altered germinal center dynamics play a role in RA pathogenesis. In contrast, only RA-a patients show an increased proportion of circulating plasmablasts.

## Introduction

Rheumatoid Arthritis (RA) is a systemic autoimmune condition characterized by chronic joint inflammation. B cells actively participate in its pathogenesis not only through the production of high-affinity and class-switched autoantibodies [1–3]: alterations in the capacity of B cells to present antigen, secrete cytokines, and modulate T cell function, are also implicated in RA [1].

Follicular helper T cells (Tfh), a major subset of effector T cells, promote B cell maturation and antibody production [4–9]. They are characterized by the expression of the transcription factor BCL-6, by their surface phenotype (CD4 + CXCR5 + ICOS + PD-1+) and their cytokine profile (IL-21, IL-10, IL-17) [4–10]. Tfh cells play an important role in the pathogenesis of autoimmunity [11], and increased numbers have been described in murine models of systemic lupus erythematosus (SLE) [11–13] and inflammatory arthritis [14]; in fact, strategies directed at reducing Tfh cell generation in these animal models seem to ameliorate disease manifestations [13,15].

Classical Tfh cells are located in secondary lymphoid organs [4–9], which prevents their routine study in human patients. Several reports have subsequently described circulating populations of CD4 T cells that express CXCR5 and display both phenotypic and functional features of true Tfh cells [16–20]. Increased frequencies of circulating Tfh cell counterparts (cTfh) have been associated with autoimmune diseases such as SLE [16,20], Sjögren’s Syndrome [21], autoimmune thyroiditis [22], chronic active hepatitis [23] and myasthenia gravis [24]. To date, only a few articles on cTfh cells in RA have been published and results are discordant among them [20,25–28].

More recently, phenotypic and functionally distinct subpopulations of cTfh cells have been described, according to the differential expression of the chemoquine receptors CXCR3 and CCR6 on CD4 + CXCR5+ T cells [17]. An altered balance of these cTfh subsets is associated with autoimmunity in children with juvenile dermatomyositis [17] and in adult patients with SLE [29] but to our knowledge has not been investigated in RA.

Therefore, our objective was to study the frequency of cTfh and cTfh cell subsets together with the frequency of circulating plasmablasts (CD19 + CD20-CD38^high^ B cells), in patients with RA. We observed that both RA patients with active disease and RA patients with inactive disease demonstrate an increased frequency of cTfh (CD3 + CD4 + CXCR5 + ICOS+) together with an overrepresentation of cTfh subsets bearing a phenotype associated with B cell helping capacity (CD3 + CD4 + CXCR5+ CCR6 + CXCR3- and CD3 + CD4 + CXCR5 + CCR6-CXCR3-). In contrast, only RA patients with active disease show an increased proportion of circulating plasmablasts.

## Methods

### Ethics statement

The study was approved by the Hospital La Paz - IdiPAZ Ethics Committee, and all subjects provided written informed consent according to the Declaration of Helsinki.

### Patients

Peripheral blood was obtained from 34 RA patients with established disease and from 34 age- and sex-matched healthy controls (HC). RA patients fulfilled at least four 1987 American College of Rheumatology criteria [30], and were receiving non-biological disease-modifying anti-rheumatic drugs (DMARDs) with or without low-dose prednisone. Among RA patients, 17 were in remission as defined by a disease activity in 28 joints (DAS28) score <2.6 (RA-r) and 17 had active disease defined by a DAS28 score >2.6 (RA-a) [31]. All patients tested positive for either rheumatoid factor (RF) or anti-citrullinated peptide antibodies (ACPA), and all of them were receiving methotrexate. Clinical data are summarized in Table 1.

**Table 1:** **Clinical characteristics of RA-a and RA-r patients**

**Variable**	**RA-a (n = 17)**	**RA-r (n = 17)**
Age, years, median (IQR)	57 (54 to 63.5)	53.5 (44 to 57.5)
Female, n (%)	16 (94)	13 (76.5)
Disease duration, years, median (IQR)	12.5 (6 to 20)	10 (6 to 16.5)
Disease activity in 28 joints (DAS28) score, median (IQR)	3.4 (3 to 4.4)	2 (1.7 to 2.4)
Rheumatoid factor-positive, n(%)	16 (94)	16 (94)
Anti-citrullinated peptide antibodies-positive, n (%)	14 (82)	14 (82)
Methotrexate, n (%)	17 (100)	17 (100)
Prednisone ≤5 mg/day, n (%)	11 (65)	4 (24)
Leflunomide, n (%)	2 (12)	3 (18)
Hydroxychloroquine, n (%)	2 (12)	4 (24)

RA-a: RA patients with active disease (DAS28 >2.6); RA-r: RA patients in remission (DAS28 <2.6) [31].

### Isolation of CD4+ T cells and B cells from human peripheral blood

Peripheral blood mononuclear cells (PBMCs) were separated immediately after blood sample collection, by Ficoll-Hypaque (GE Healthcare Biosciences AB, Uppsala, Sweden) density gradient centrifugation. CD4+ T or B cells were purifed from freshly isolated PBMCs by exhaustive immunomagnetic negative selection in an Automacs (Miltenyi Biotec, Bergisch Gladbach, Germany), using the CD4+ T Cell Isolation Kit or the B Cell Isolation Kit II from Miltenyi Biotec. Isolated CD4+ T cells or CD19+ B cells were >98% pure. CD4 + CXCR5+ were selected from total CD4+ T cells using PE-labeled CXCR5 microbeads (Miltenyi Biotec). Subsequently, cTfh subpopulations were sorted from CD4 + CXCR5+ T cells in a FACSVantage SE flow cytometer (BD Biosciences, San Jose, CA, USA) after staining isolated CD4 + CXCR5+ T cells with CXCR3 and CCR6. Sorting was directed to isolating CXCR5 + CXCR3 + CCR6- T cells (Tfh-Th1 cells), CXCR5 + CXCR3-CCR6- T cells (Tfh-Th2 cells) and CXCR5+ CXCR3-CCR6+ T cells (Tfh-Th17 cells).

Naïve (CD19 + CD27-) B cells were selected from total CD19+ B cells by negative selection using CD27+ microbeads (Miltenyi Biotec). T and B cell subpopulations were >98% pure and used immediately after isolation.

### B cell/ T cell co-cultures

To assess the functional capacity of cTfh subpopulations, sorted peripheral blood Tfh-Th1, Tfh-Th2 or Tfh-Th17 cells (5 × 10^4^ cells/well), were co-cultured for 13 days with autologous naïve B (CD19 + CD27-) cells (5 × 10^4^ cells/well) in U-bottom 96-well plates containing RPMI 1640 medium (Lonza, Alendale, NJ, USA) with 10% FCS, 2 mM L-glutamine, 50 U/ml penicillin, 50 μg/ml streptomycin and 50 μM 2-mercapto-ethanol (complete RPMI medium). Endotoxin-reduced staphylococcal enterotoxin B (SEB) (1 μg/ml) (Sigma-Aldrich, St Louis, MO, USA) was also added to the cultures, as the Ig production in Tfh/B cell cocultures has been shown to depend on cognate T/B cell interactions [16]. Concentrations of IgG, IgA and IgM were measured in co-culture supernatants by ELISA.

### Flow cytometry

The frequency and phenotype of Tfh-like cells and plasmablasts present in the peripheral blood of RA patients and HC was assessed by flow cytometry in a FACSCalibur flow cytometer using CellQuest software (BD Biosciences), after staining freshly isolated PBMCs with antibodies directed against surface phenotypic markers. Fluorochrome-conjugated mAbs from BD Pharmingen (San Diego, CA, USA) were used to examine the expression of CD3, CD4, CD8, CXCR5, ICOS, PD-1, CCR6, CXCR3, CD19, CD20, and CD38.

### ELISA

Cell-free co-culture supernatants were collected and stored at −80°C. The concentrations of immunoglobulins were measured by ELISA. In brief, 96-well plates (MaxiSorp, Thermo Fisher Scientific, Waltham, MA, USA) were coated overnight at 4°C with 10 μg/ml mouse monoclonal anti-human IgG, IgA or IgM (AbD Serotec, Munich, Germany), and subsequently blocked with 2% BSA/PBS. Standard curves of human IgG, IgA or IgM (Sigma-Aldrich) together with culture supernatants diluted in 2% BSA/PBS were incubated for 3 hours at room temperature, washed and developed with horseradish peroxidase-conjugated goat anti-human IgG, IgA or IgM (AbD serotec) followed by TMB substrate solution (Pierce Biotechnology, Rockford, IL, USA). Absorbance was measured at 450 nm in a Synergy H4 Hybrid Multi-Mode Microplate Reader (BioTec Instruments, Inc., Winoosi, VT, USA).

### Statistical analysis

Comparison between groups was by Mann-Whitney or Kruskal-Wallis test. When appropriate, Bonferroni correction for multiple comparisons was applied. Correlations were analyzed using Spearman’s rank correlation coefficient. All analyses were performed using Prism version 5.0 software (GraphPad Software, San Diego, CA, USA).

## Results

### Functional capacity of cTfh subpopulations

**Figure 1:**
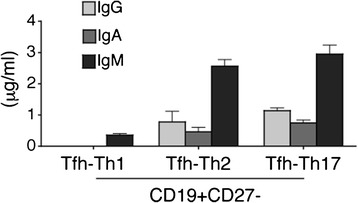
**Functional capacity of circulating CD4 + CXCR5+ T cell subsets.** CD4 + CXCR5+ T cell subsets (Tfh-Th1, Tfh-Th2 or Tfh-Th17) isolated from peripheral blood of HC were co-cultured with autologous CD19 + CD27- naïve B cells for 13 days. Shown are concentrations of IgG, IgA and IgM in 13-day co-culture supernatants. Line and bar graphs represent the mean and SD of five independent experiments. Tfh-Th1 cells, CXCR5 + CXCR3 + CCR6- T cells; Tfh-Th17 cells, CXCR5+ CXCR3-CCR6+ T cells; Tfh-Th2 cells, CXCR5 + CXCR3-CCR6- T cells.

Our first goal was to examine the functional capacity of sorted peripheral blood Tfh-Th1, Tfh-Th2 and Tfh-Th17 cells. In co-cultures with autologous Tfh-Th2 or Tfh-Th17 cells, naïve B cells secreted both IgG and IgA, together with IgM (Figure 1). In contrast, naïve B cells co-cultured with Tfh-Th1 cells did not produce detectable IgG or IgA but only low amounts of IgM (Figure 1). This indicates that among circulating CXCR5+ T cells, only Tfh-Th2 and Tfh-Th17, but not Tfh-Th1 cells, are able to induce maturation of naïve B cells, which is consistent with results published by Morita *et al*. [17].

### An increased frequency of circulating Tfh counterparts is apparent in the peripheral blood of RA patients with active or inactive disease

**Figure 2:**
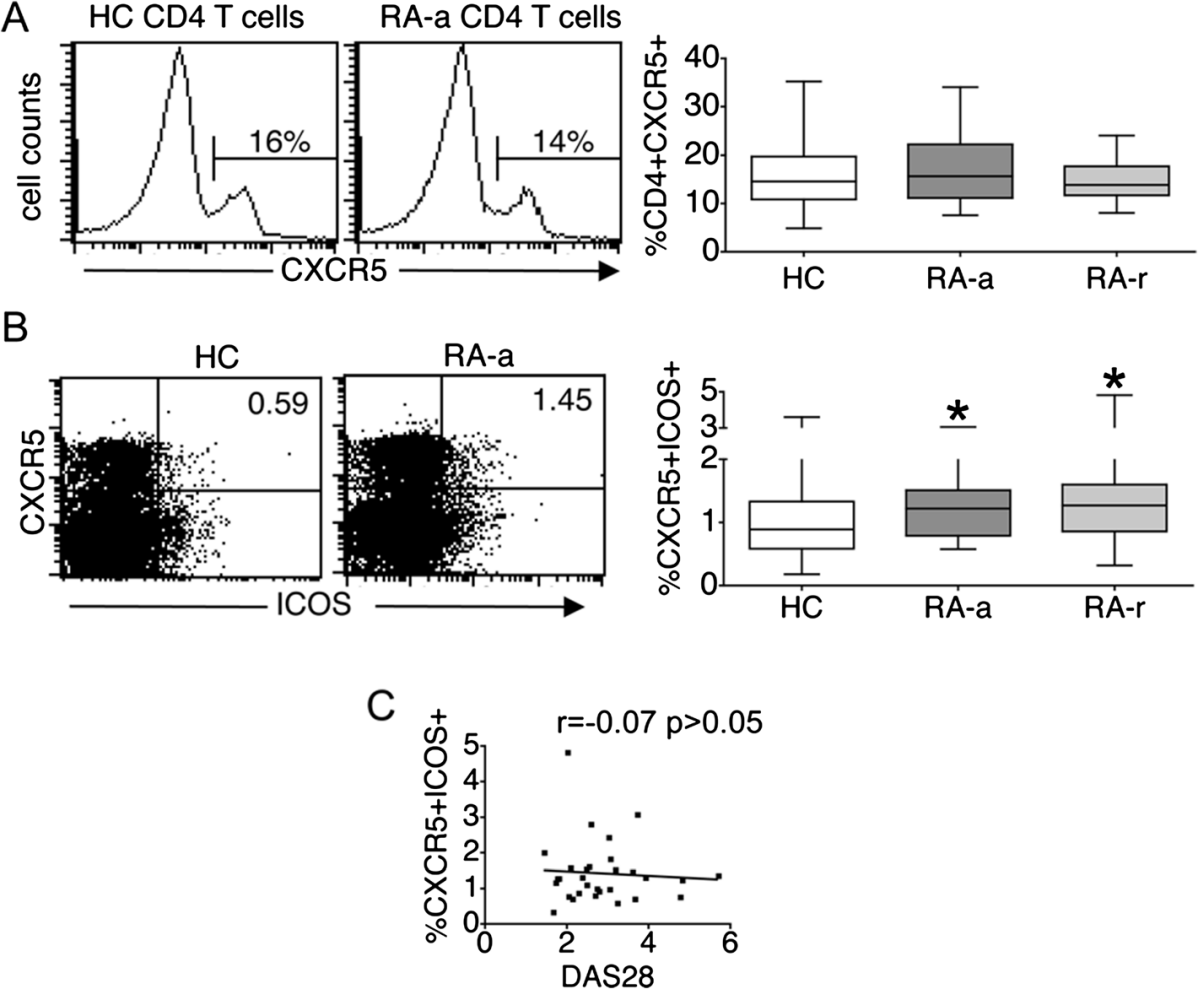
**Frequency of circulating follicular helper T cells (cTfh) in patients with rheumatoid arthritis (RA). (A)** The frequency of circulating CD4 + CXCR5+ T cells was not different in RA patients with active (RA-a) or inactive disease (RA-r) as compared with controls (HC). Histograms represent CXCR5 expression in cells gated for CD3 and CD4. **(B)** Increased frequency of circulating Tfh counterparts in RA-a or RA-r patients, defined as CD4 + CXCR5 + ICOS+ T cells. Representative dot plots demonstrate ICOS and CXCR5 expression in cells gated for CD3 and CD4. **(C)** Relationship between cTfh proportions and disease activity as determined by disease activity in 28 joints (DAS28) score [31]. In **A** and **B**, box and whiskers plots represent the median, interquartile range, maximum and minimum values calculated from the 17 RA-a patients, 17 RA-r patients and 34 HC that were studied. *P <0.01 versus HC.

We then sought to examine the expression of Tfh phenotypic surface markers on CD4+ T cells present in the peripheral blood of RA-a or RA-r patients. Importantly, the absolute numbers of circulating CD4+ T cells were not different among the three studied groups (mean ± standard error of the mean (SEM), 1,141.0 ± 142.8 × 10^3^ CD4+ T cells/ml in RA-a versus 950.6 ± 132.3 in RA-r, versus 1,024.2 ± 163.8 in HC, *P* >0.1). In addition, the frequency of CXCR5+ cells among circulating CD4 + T lymphocytes was not different in RA-a or RA-r patients as compared with HC (Figure 2A). In contrast, the frequencies of circulating CD4 + CXCR5 + ICOS+ T cells, which are considered as circulating counterparts of classical Tfh cells [16,18,19], were significantly increased both in RA-a and RA-r patients (Figure 2B). Among RA patients, there was no correlation between disease activity parameters and frequency of cTfh (Figure 2C).

### RA patients with active or inactive disease demonstrate an altered balance of cTfh subsets

**Figure 3:**
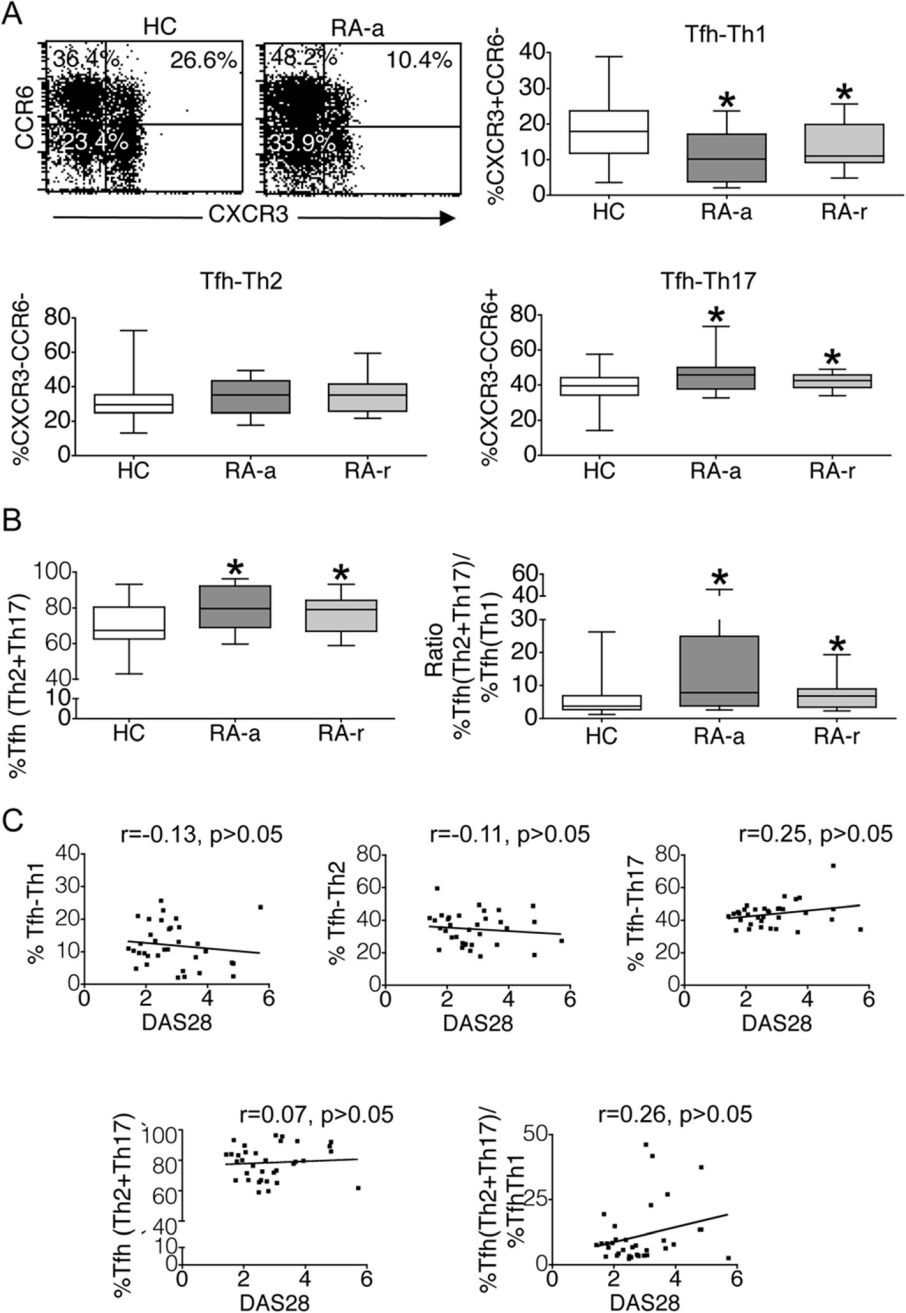
**Frequency of circulating follicular helper T cell (cTfh) subsets in patients with rheumatoid arthritis (RA). (A)** Frequency of circulating Tfh-Th1, Tfh-Th2 and Tfh-Th17 cells in R-a, RA-r and HC. Representative dot plots demonstrate CXCR3 and CCR6 expression in cells gated for CD3, CD4 and CXCR5. **(B)**. Overabundance of Tfh-Th2 and Tfh-Th17 cells over Tfh-Th1 cells in RA-a and RA-r patients. **(C)** Relation of cTfh subset proportions or ratio with disease activity as determined by disease activity in 28 joints (DAS28) score [31]. *P <0.01 versus HC. Tfh, Follicular helper T cells; Tfh-Th1 cells, CXCR5 + CXCR3 + CCR6- T cells; Tfh-Th17 cells, CXCR5+ CXCR3-CCR6+ T cells; Tfh-Th2 cells, CXCR5 + CXCR3-CCR6- T cells.

RA patients with active or inactive disease demonstrated a decreased frequency of circulating Tfh-Th1 cells together with an increased frequency of Tfh-Th17 cells, whereas the frequency of Tfh-Th2 cells tended to be elevated but was not statistically different from HC (Figure 3A). Furthermore, the sum of %Tfh-Th2 plus %Tfh-Th17 cells, and the ratio (%Tfh-Th2 + %Tfh-Th17)/%Tfh-Th1 were significantly higher in RA-a or RA-r patients as compared with HC (Figure 3B).

That is, both RA-a and RA-r patients demonstrated a relative overabundance of Tfh cell subsets bearing a B cell helper phenotype. Among RA patients, there was no correlation between disease activity parameters and the observed proportions of cTfh subset or cTfh subset ratios (Figure 3C).

### RA patients with active disease demonstrate an increased frequency of circulating plasmablasts

**Figure 4:**
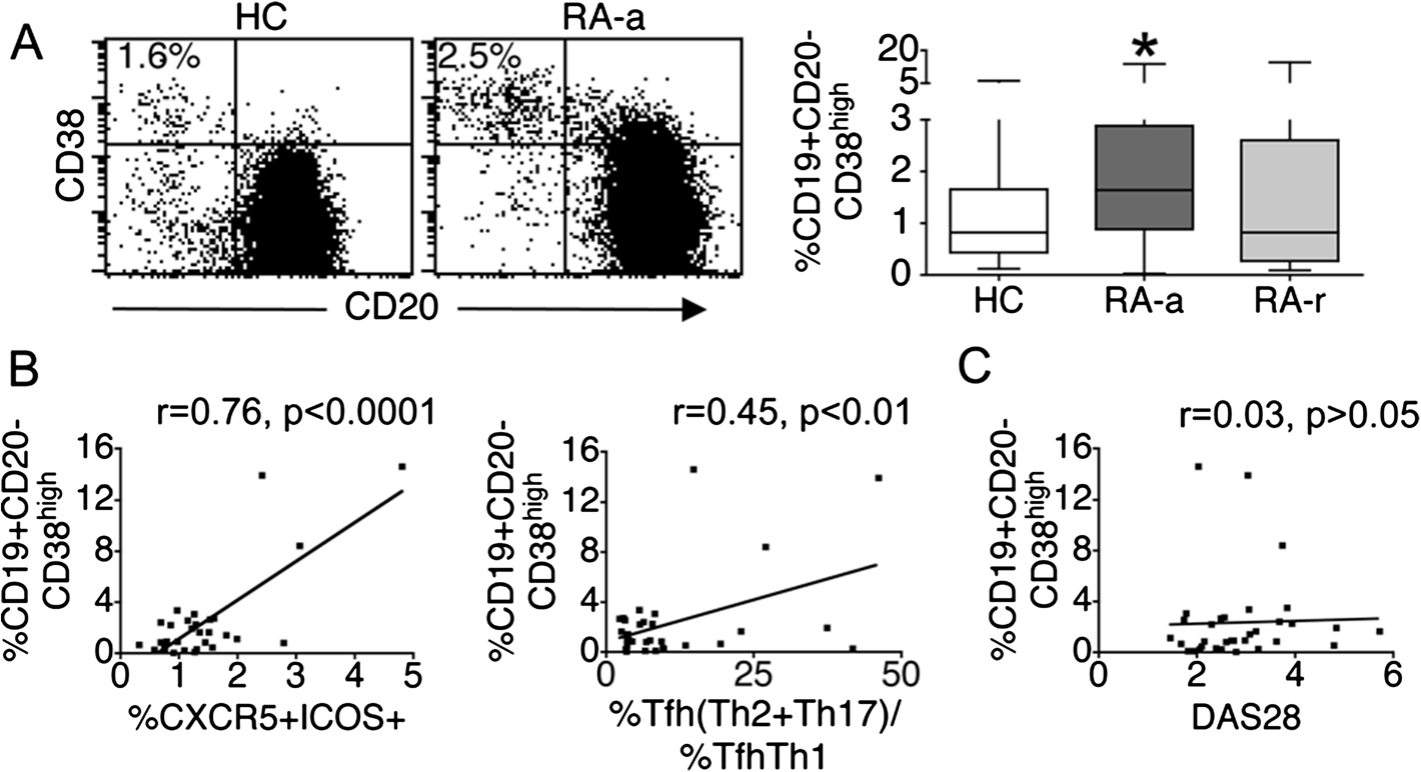
**An increased frequency of circulating plasmablasts in rheumatoid arthritis patients with active disease (RA-a) is correlated with the frequency of circulating follicular helper T cells (cTfh). (A)** Patients RA-a but not with rheumatoid arthritis in remission (RA-r) demonstrate an increased frequency of circulating plasmablasts. Shown are representative dot plots of CD20 and CD38 expression on cells gated for CD19 (left panel), together with box and whisker plots representing the median, interquartile range, maximum and minimum values calculated from 17 RA-a patients, 17 RA-r patients and 34 healthy controls (HC) (right panel). *P <0.01 versus HC. **(B)** The frequency of circulating plasmablasts in RA-a patients is positively correlated with the frequency of circulating Tfh counterparts and with the ratio (%Tfh-Th2 + %Tfh-Th17)/%Tfh-Th1 cells. **(C)** Relation of circulating plasmablast frequencies with disease activity as determined by disease activity in 28 joints (DAS28) score [31].

The absolute number of circulating CD19+ cells was not different in RA patients with active or inactive disease as compared with controls (mean ± SEM, 86.65 ± 13.06 × 10^3^ CD19+ cells/ml in RA-a versus 87.51 ± 15.78 in RA-r patients versus 86.70 ± 9.49 in HC). At the same time, the frequency of circulating plasmablasts, defined as CD19 + CD20-CD38^high^ B cells, was increased in RA-a but not in RA-r patients (Figure 4A). Interestingly, in RA patients, the frequency of circulating plasmablasts was positively correlated not only with the frequency of circulating Tfh counterparts but also with the ratio (%Tfh-Th2 + %Tfh-Th17)/%Tfh-Th1 cells, (Figure 4B). Among RA patients, there was no correlation between disease activity parameters and proportions of circulating plasmablasts (Figure 4C).

## Discussion

The association of RA with high-affinity, class-switched autoantibodies [1–3], indicates the implication of B helper T cells in disease pathogenesis and therefore we deemed it interesting to study cTfh biology in this condition.

It is herein described that among circulating CD4 + CXCR5+ T cells, only sorted Tfh-Th2 and Tfh-Th17 but not Tfh-Th1 cells, display functional properties of classical Tfh present in the germinal centers of secondary lymphoid organs: Tfh-Th2 and Tfh-Th17 but not Tfh-Th1 cells, had the capacity to promote maturation and induce isotype switching of naïve B cells, as previoulsy reported by Morita *et al*. [17]. In addition, we observed that not only RA patients with active disease but also RA patients in remission, demonstrate an increased frequency of cTfh defined as CD4 + CXCR5 + ICOS+ T cells [16,18,19], together with an overrepresentation of cTfh subsets bearing a B cell helper phenotype (Tfh-Th2 and Tfh-Th17). The augmented CD4 + CXCR5 + ICOS+ T cell proportions are not attributable to the presence of activated T cells in peripheral blood; although CXCR5 is typically upregulated upon *in vivo* T cell activation [32], total CD4 + CXCR5+ T cells themselves were not increased in our RA patients with active or inactive disease.

Therefore, this constitutive overabundance of cTfh in RA seems related to disease phenotype and pathogenic mechanisms but not to RA disease activity, as has been reported in patients with SLE [16]: Simpson *et al*. described an expansion of cTfh in a subset of SLE patients with severe disease, which does not vary with time, modifications in disease activity or treatment [16]. In this context, it has been proposed that increased numbers of cTfh can be a signature of human immune-mediated diseases [19]. In fact, a causal relation between accumulation of Tfh cells and autoimmunity has been demonstrated in mice homozygous for the san allele of Roquin (Roquin^san/san^), a protein regulator of mRNA stability [33]. Roquin represses ICOS mRNA post-transcriptionally, and homozygosity for its san allele mediates ICOS overexpression and generation of Tfh cells [13,33]. Roquin^san/san^ mice demonstrate increased numbers of Tfh cells together with aberrant GC formation and positive selection of pathogenic high-affinity autoantibodies, resulting in a lupus-like phenotype [13,33]. Furthermore, abrogating Tfh cell generation prevents lupus development in the Roquin^san/san^ mice, and transfer of Tfh cells from these mice induces the spontaneous formation of GCs [13].

Conversely, patients with loss-of-function mutations in CD40L, NEMO, STAT3, IL-21R or BTK and patients with gain-of-function mutations in STAT1, demonstrate a severely impaired generation of germinal centers together with decreased circulating CD4 + CXCR5+ T cells [34], reinforcing the notion that cTfh numbers are a reflection of the pool of classical Tfh in lymphoid organs.

The recent description by Morita *et al*. of three different subsets of circulating CD4 + CXCR5+ T cells with distinct functional properties [17] sheds new light into the biology of Tfh and their pathogenic implication in autoimmune conditions. Of note, it has been reported that among circulating Tfh cells, the Tfh-Th17 subset is most significantly reduced in all immune deficiencies above mentioned [34], indicating the importance of these cells as B cell helpers. In this context, Tfh-Th17 was the most significantly elevated Tfh subset in our RA patients, which further suggests their leading role in promoting autoantibody production.

Of note, altered proportions of circulating Tfh subsets in patients with juvenile dermatomyositis (JDM) seem to be associated with disease activity [17], which contrasts with findings in our RA patients. This suggests that the mechanisms leading to an altered regulation of Tfh cell subset differentiation may be different in JDM and RA.

Interestingly, we observed that whereas RA-a patients show an increased frequency of circulating plasmablasts, the frequency of circulating plasmablasts in RA-r was not different from controls. This parallels reported observations indicating that response to treatment in RA is associated with a reduction in the titers of RF and/or ACPA, whereas RA patients who do not respond to treatment maintain antibody levels comparable to those found at baseline [35,36]. Of note, despite a signficant reduction of RF and/or ACPA levels associated with therapeutic response, seropositive RA patients rarely become seronegative, indicating the persistence of a constitutive autoimmune alteration [35,36].

A reason for the finding of normal circulating plasmablasts in the presence of elevated cTfh proportions in RA-r patients is not readily apparent to us. We can only speculate that, in patients who respond and achieve remission, treatment with non-biological DMARDs may block the action of constitutively increased Tfh cell numbers on B cell biology, thereby preventing the generation of increased proportions of plasmablasts. Conversely, in RA patients who do not achieve a complete response to DMARDs, these drugs would not be able to modify the function of Tfh. Alternatively, plasmablast generation through the extrafollicular pathway could account for the discordance between cTfh and plasmablast numbers in patients with active or inactive disease [37,38].

## Conclusions

In summary, we have described a constitutively increased frequency of cTfh together with an overrepresentation cTfh subsets bearing a B cell helper phenotype in RA. This indicates that excessive Tfh cell generation and altered germinal center dynamics may play a role in the pathogenesis of this condition. In addition, it points to modifications of Tfh effector molecules or Tfh subset balance as future therapeutic targets in RA.

## Abbreviations

ACPA: anti-citrullinated peptide antibodies
BSA: bovine serum albumin
cTfh: circulating Tfh
DAS28: disease activity in 28 joints
ELISA: enzyme-linked immunosorbent assay
FCS: fetal calf serum
HC: healthy controls
mAb: monoclonal antibody
PBMC: peripheral blood mononuclear cell
PBS: phosphate-buffered saline
RA: rheumatoid arthritis
RA-a: patients with active rheumatoid arthritis
RA-r: patients with rheumatoid arthritis in remission
RF: rheumatoid factor
SEM: standard error of the mean
SLE: systemic lupus erythematosus
Tfh: Follicular helper T cells
Tfh-Th1 cells: CXCR5 + CXCR3 + CCR6- T cells
Tfh-Th17 cells: CXCR5+ CXCR3-CCR6+ T cells
Tfh-Th2 cells: CXCR5 + CXCR3-CCR6- T cells
